# Mental Health Challenges at the Intersection of First-Year, First-Generation College Students and Second-Generation Immigrant Identities: A Qualitative Study

**DOI:** 10.3390/healthcare14010021

**Published:** 2025-12-21

**Authors:** Cassandre Horne, Precious Chibuike Chukwuere

**Affiliations:** Christine E. Lynn College of Nursing, Florida Atlantic University, 777 Glades Rd., Boca Raton, FL 33431, USA

**Keywords:** first-generation college students, short-story research, second-generation immigrants, mental health, socio-ecological model

## Abstract

**Background/Objectives:** First-year, first-generation college students who are also second-generation immigrants often face significant mental health challenges as they navigate both higher education and early adulthood. This study explored how mental health challenges are shaped by their intersecting identities and framed their experiences using Bronfenbrenner’s socio-ecological model. **Methods:** This study was conducted in the office of first-generation success at a 4-year R1 university, adopting a qualitative research approach and a small stories research design. A purposive sampling technique was implemented to sample first-year, first-generation students and second-generation immigrants. Two focus group discussions were conducted, each with groups comprising 11 participants (*n* = 22). The participants were between 18 and 19 years old. The data were analyzed using a thematic approach, with trustworthiness ensured through the establishment of credibility, dependability, confirmability, and transferability. **Results:** Two themes emerged: “Finding self” and “Balancing Competing Demands” within the first-year, first-generation population. Additionally, stress was identified in the second-generation immigrant group under the theme of “Cultural Expectations”. **Conclusions:** Framing the stories within the socio-ecological model illustrates the multi-layered mental health burden of this population group, particularly within the socio-political climate shaped by heightened immigration policy, restrictive enforcement practices, and public discourse surrounding immigrant communities. Recognizing their mental health as integral to their overall health and academic success highlights the need to broaden scholarly and clinical understanding of individuals and compounding contextual variables that may be related to adverse emotional states.

## 1. Background

The transition to post-secondary education places first-year students at heightened risk for psychological challenges [1]. The onset of emerging adulthood, a life stage between adolescence and adulthood, can exhibit distinctive developmental characteristics. This includes transitions from living with to living apart from their parents, pursuing education or training, and entering the workforce, all while navigating identity exploration and feelings of instability. This is a critical time for the onset of several mental health problems, including anxiety and depression [2]. Anxiety and depression are the most frequently reported conditions among first-year college students [3]. Anxiety and depression are often experienced when college students face compounded stressful events without adequate support, with first-year students being especially vulnerable due to the transition into college [1]. Furthermore, this transition may also be particularly challenging for students identified as high-risk due to specific demographic factors (e.g., racial or sexual minority, first-generation, international, etc.) [1] (p. 1). These transitional experiences, navigating autonomy, identity exploration, building social connections, and establishing independence, are closely linked to mental health outcomes, as challenges in adapting to new responsibilities can heighten stress, anxiety, and vulnerability to depression during this critical period [2]. This stage underscores a critical phase of psychological development and self-identification, marked by increased vulnerability to emotional imbalance, stress, and anxiety. It is important not to conflate this experience with extended adolescence, as doing so would underestimate the capacities for self-direction, self-reflection, decision-making, and independent living [1,4]. These changes necessitate the development of coping skills and decision-making capabilities, which are essential for achieving success in their academic pursuits while managing emotional exhaustion and/or economic burden.

For first-year, first-generation college students (FGCSs), those who are the first in their families to attend college, these challenges are exacerbated by the stress of navigating an unfamiliar academic environment with minimal knowledge or guidance from family. FGCS make up one-third of the undergraduate student population in the United States [3], and most of the time, they depend heavily on institutional support to compensate for inadequate economic and social resources [5]. In addition, feelings of not belonging along with the need to prioritize employment are common among low-income and minoritized students, further exacerbating their challenges [3,5]. Such stressors can exacerbate isolation and self-doubt, contributing to mental health difficulties. According to the National Institute of Mental Health 2021–2022 [6] data, the prevalence of adults with a major depressive episode was highest among individuals aged 18–25 (18.6%). Although this statistic reflects the broader young adult population, minoritized student groups inclusive of FGCSs may face heightened academic, financial, and social pressures that place them at even greater risk, underscoring the importance of specifically examining their mental health needs.

Similarly, second-generation immigrants (SGIs), those born in the United (U.S.) States to immigrant parents, face unique challenges particularly when cultural expectations at home differ from those in mainstream society. The U.S. is home to more migrants than any other country worldwide [7], and children of immigrants now represent one of the fastest-growing population groups [8]. SGIs navigate bicultural expectations while balancing family-prescribed values with social norms [9]. For example, SGIs often experience pressure to pursue “practical” careers such as medicine or law, which can exacerbate mental health strain even as they try to assert personal aspirations [9]. Transitioning into adulthood can be characterized by moving away from home to attend school, financial independence, exploring career options, and forming lifelong relationships. However, SGIs must consider their families’ cultural expectations, as their newfound trajectory in career pursuits serves as an example for future generations. This pressure can create an environment for significant mental health strain. Identity confusion, cultural stigma, emotional exhaustion, depression, and anxiety may result from the tension between personal aspirations, feelings of cultural obligation, and familial expectations [10]. This population can often feel entangled between two cultural dichotomies, struggling to belong to either. Rad et al. (2023) and Hinson and Weiser (2025) provide evidence that these competing expectations contribute to psychological strain, including identity confusion, emotional exhaustion, and increased vulnerability to mental health challenges [10,11]. Other challenges, such as acculturation stress, may arise due to the pressure to succeed, which extends not only to the individual but also to the entire family [9,12]. In these situations, mental health emerges as a critical area of concern, particularly for a population whose experiences are shaped by cultural expectations.

High levels of stress and accumulated stress during this developmental transition into adulthood are of critical importance, as evidence suggests that life stressors are inversely related to psychological well-being among undergraduate students [2]. Despite overlapping stressors, there is limited prior research that has examined how FGCS and SGI identities intersect to compound mental health strain, leaving a gap in understanding the unique challenges faced by students at this intersection. More specifically, there is a notable gap in qualitative research that explores how these students make meaning of their lived experiences at this intersection, an absence that limits scholarly understanding of nuanced emotional, cultural, and contextual factors shaping their mental health. Due to each population group (FGCSs and SGIs) facing several challenges independently during a high-stress transitional time period, the health literature must examine the mental health of this intersecting identity. Thus, addressing mental health challenges continues to be at the forefront of healthcare discourse. This study aims to investigate the mental health strain within the identity intersection of first-year, first-generation college students and second-generation immigrants, while also contextualizing their experience within the socio-ecological model. The research questions that guided this study are as follows:

Q1. What psychological or emotional challenges do first-year, first-generation college students encounter in their pursuit of higher education?

Q2. How do cultural expectations uniquely shape mental health stressors for FGCS-SGIs?

## 2. Theoretical Framework

This study was grounded in Story Theory and operationalized in the Socio-ecological model (SEM). Story Theory served as the interpretive lens for understanding how students connected their past, present, and anticipated future selves while articulating moments of tension, struggle, and movement toward ease. Consistent with the small stories research design, participant narratives were approached not as objective accounts of events but as situated constructions shaped by context, identity, cultural expectations, and interactional dynamics. Meaning was therefore understood as emergent, relational, and produced in the moment of telling. This epistemological stance informed all aspects of the study, including data collection, interpretation, and the use of Story Theory as a lens for understanding how participants connect past, present, and future experiences through narrative.

Story Theory is a nursing theory that positions stories as the fundamental dimension of human experience [13]. One of the assumptions is that people live in an expanded present moment, where past and future events are transformed into the present [14]. This is in line with constructivist epistemology, which assumes that knowledge is co-constructed through social interaction, dialogue, and meaning making. Story Theory aligns directly with the developmental and psychosocial realities of FGCS and SGI students. This population navigates an expanded reality as they attempt to reconcile their new identities with the familiar nuances of their past in an environment that is actively transforming their future. The three concepts of this theory (connecting with self-in-relation, intentional dialogue, and creating ease) guided its application as interpretive anchors in this study. Through intentional dialogue, participants engaged with their emotions in an effort to move towards ease by sharing their stories.

FGCS-SGIs frequently engage in relational identity work, negotiating their sense of self in relation to family expectations, community norms, and academic environments. A story represents the narration of events as remembered, infused with unique personal perspectives that reveal thoughts and feelings, shape meaning, and guide choices in the present [13]. The discussion will be further contextualized within the socio-ecological model (SEM). A socio-ecological model for mental health recognizes that the individual’s well-being is shaped by the dynamic interplay of personal characteristics, psychological factors, close relationships, community environment, and broader societal factors [15,16]. This model provides a comprehensive look at the varied and complex influences on mental health for those with intersecting minority identities. An SEM displays where pressures originate and how these pressures interact across multiple levels of a person’s environment, providing analytic layers such as the individual, micro-, meso-, exo-, and macro-systems.

The microsystem refers to the immediate environment closest to an individual, encompassing interactions and relationships that exert the most direct influences. The mesosystem refers to the interactions between multiple microsystems (such as home–school or peer–family interactions), creating linkages that shape the individual’s experience across various settings, rather than representing environments that directly contact the individual. The mesosystem (beyond immediate interactions) includes interactions and environments that have contact with the individual, such as schools and neighborhoods [17]. This includes interactions between micro-level systems [16]. The exosystem does not directly impact the individual but exerts both positive and negative interactive forces, such as community and social networks [17]. The macro level encompasses societal, religious, and cultural values and beliefs shaped by the social environment, influencing the expectations and opportunities available [16].

The socio-ecological model provided the contextual scaffold for interpreting how these narrative meanings were shaped by multi-level influences, including family expectations, peer relationships, institutional factors, and broader cultural norms. Incorporating students’ personal meaning-making processes (Story Theory) with the multi-level contextual forces that shape their mental health (socio-ecological model), offers a more holistic understanding of their lived experiences. Together, these frameworks offered an aligned analytic structure that illuminated how participants made sense of these moments through personal meaning-making processes. This integration informed the development of categories, guided themed refinement, and supported conclusions that accounted for both the intimate narrative processes and the broader environmental forces shaping participants’ mental load.

## 3. Methodology

This study adopted a qualitative research approach and a small stories research design. A small stories research design is a model of narrative inquiry that focuses on the everyday, fragmented stories of people [18,19]. A small stories research design was critical for this study because it served as the methodological foundation, guiding how participants’ narratives were elicited, attended to, and analyzed as fragmented, interactional story moments situated in their everyday lives. It directed attention to the lived, momentary construction of meaning within the fragmented narratives of our participants’ lives. This approach was initially developed to address the proliferation of fragmented storytelling phenomena in everyday interactional environments [20] (p. 265). Small stories frequently emerge as counter-stories that do not fit expectations of who the tellers should be and what stories they tell, in these cases introducing contradictions, dilemmas, and tensions on the part of the tellers [20].

### 3.1. Sample and Recruitment

This study took place in the Office of First-Generation Student Success at a 4-year R1 university. This office supports FGCSs through various resources, including dedicated academic support personnel, referrals, scholarships, advising, and programming designed to promote the success of first-generation students. Recruitment flyer announcements were placed at the office. There were 22 participants (9 males and 13 females), aged 18 to 19, who were first-generation college students in their first year of college. A sample size of 22 participants is methodologically appropriate for a qualitative research approach and a small stories design for two reasons. First, a qualitative research approach and a small stories design prioritizes the depth of meaning-making over numerical representation; therefore, adequacy is determined by information richness rather than statistical sufficiency [20,21,22]. Second, a qualitative research approach and a small stories design specifically analyzes short, fragmented, and interaction-generated narratives, allowing multiple distinct story units to emerge from each participant [20,21,22]. With 22 students across two highly interactive focus group discussions, the dataset contained dozens of story segments, contradictions, tensions, and meaning-making episodes, exceeding what is required for conceptual saturation.

The office of First-Generation Student Success also confirmed that participants were first-year, first-generation college students served by their office, with half of the participants self-identifying as second-generation immigrants from diverse cultural backgrounds. The demographic breakdown is in Table 1. The students contacted the first author by way of the information presented on the flyer for further information regarding this study. This was also to ensure that participants voluntarily elected to participate in the study. Recruitment was supported through the Office of First-Generation Student Success, and the study was approved by the Institutional Review Board. The authors had no prior affiliation with the students served by this office and were not involved in their educational or support programming, minimizing the potential for coercion or undue influence.

Informed consent was obtained from all participants prior to data collection. Prior to obtaining informed consent from the participants, their rights regarding participation in the study were explained to them, and associated written consent documents were provided. Participants were informed that they could pull out of the study at any time without consequence. They were allowed and encouraged to ask questions regarding their participation in the study, and appropriate answers were provided, enabling them to make informed decisions about participating. Participants received a one-time compensation of a USD 20 gift card for their time and participation.

### 3.2. Data Collection

Stories of FGCSs and SGIs were collected through focus group discussions. Focus group discussions were intentionally chosen over individual interviews because participants’ shared experiences, collective meaning, and social dynamics regarding the phenomenon can unfold naturally in group interaction, particularly among peers with shared identities. Focus group discussions offer an interactive environment that allows participants to become more at ease by building on one another’s insights, validating common challenges, and articulating experiences that may not emerge in individual interviews [23,24,25]. In addition, focus group discussions enable a roundtable discussion regarding a given topic, fostering the gathering of broader and in-depth data from participants, as they often recall additional details when listening to their peers. Before the commencement of the focus group discussion session, participants were informed of their rights regarding participation in the study, which included the right to withdraw from the study at any time without any negative consequences. They were allowed to ask questions regarding these rights, with responses provided by the focus group discussion facilitator. Participants were encouraged to share their experiences related to the phenomenon—particularly meaningful life events, experiences, or concerns. The stories served as a conduit to understanding the mental health challenges of first-year, first-generation college students and second-generation immigrants. Operationalizing intentional dialogue involved the researcher prompting participants with broad, non-directive questions, followed by a sustained dialogue and co-creation of meaning through prompts that encouraged deeper reflection within participants stories, e.g., “Tell me more about what that meant to you.” Participants gradually became more at ease as they identified shared meanings and common experiences among their peers.

Two focus group discussions were conducted with 11 participants in each group. Within the first focus group, 5 of the 11 participants held a dual identity of SGI and 6 identified as FGCSs only. In the second focus group, 6 of the 11 participants also identified as SGIs, while 5 identified as FGCSs only. Using a Semi-structured Interview Guide (Supplementary Materials), the following main questions were asked: “What is your experience of being a first-generation college student?” “Do you have support?” “What does support look like for you?” “What weighs you down mentally and/or emotionally?” Participants also wrote down their answers to the question “what is currently causing you the most mental load or stress?” Small stories were shared, adhering to the reality that many of our stories are “messy,” developing without easily identifiable endpoints, and effective in bringing to the fore silenced, untold, devalued, and discarded stories [20].

The focus group discussion lasted between 40 min and 1 h. The focus group discussion was audio recorded. Permission to record the sessions was obtained from the participants within the consenting procedures. Participants were informed that full confidentiality is not guaranteed because, in a group setting, the facilitator cannot fully control what other participants may disclose outside the session. Hence, the following group rules were established to guide the discussion and protect confidentiality: 1. All discussions within the group remain confidential and should not be shared with anyone outside the group. 2. Group members have an obligation to respect one another’s rights and refrain from using abusive language. 3. Participants were encouraged to allow each other to express their views freely without interruption from another person. At the outset of each session, participants verbally affirmed these rules, and the facilitator reiterated that breaching confidentiality would violate the group agreement and could lead to the participant being reminded of, or withdrawn from, the group process if necessary. To ensure confidentiality of participant information, no personal identifiers were collected within the group setting. The focus group facilitator took field notes and kept a reflective journal, endeavoring to listen effectively to the participants during the discussion while maintaining control of the sessions. The focus group discussion facilitator incorporated the following skills: probing, attentive listening, flexibility, adaptability, and emotional intelligence. Throughout the data collection, the researchers continuously reflected and made explicit their positionality to foster transparency and minimize interpretive bias. Theoretical saturation was assessed across both focus group discussions. Saturation was reached when no new codes, narrative patterns, or meaning-making processes emerged during the final analytic cycles [26]. Given the richness of multiple small-story segments produced by 22 highly interactive participants, saturation was achieved following the analysis of the second focus group discussion. Despite these safeguards, the inability to enforce confidentiality among participants remains a methodological limitation of the focus group design and is acknowledged as such in this study.

### 3.3. Data Analysis

The focus group discussion data were transcribed verbatim, uploaded on ATLAS.ti 22, and analyzed using Braun and Clarke’s (2021) [22] six-phase thematic analysis framework. Small stories research [20] informed how narrative segments were identified and interpreted throughout the analysis. The following steps were followed; 1. Familiarization with the Data: Researchers read the transcript thoroughly while listening to the audio recordings to gain an in-depth understanding of the dataset. In alignment with small stories research, the transcript was segmented into small story units such as brief narrative fragments, contradictions, emotional shifts, turning points, and moments of self-positioning. Reflexive memos were documented to record early analytic impressions. 2. Generating Initial Codes: Each small story unit served as the foundation for initial coding. Codes captured narrative functions (e.g., resistance, identity negotiation, coping, tension), cultural meanings, and interactional dynamics. 3. Searching for Themes: Codes were clustered into preliminary themes by examining patterns across the dataset. For RQ1, all transcripts were analyzed as a whole sample dataset. For RQ2, a secondary analytic layer was added by comparing codes generated from the FGCS and SGI subgroups, allowing culturally specific themes to emerge without fragmenting the foundational analysis. 4. Reviewing Themes: Themes were reviewed against coded data extracts and the full dataset to ensure coherence and distinctiveness. Cross-validation was conducted; both coders independently examined theme boundaries and confirmed that each theme was grounded in the data. An analytic audit trail documented all refinements and decisions. 5. Defining and Naming Themes: Themes were refined, clarified, and named to reflect their central organizing concepts. Narrative features of small stories such as identity negotiations, cultural tensions, and turning points were incorporated into theme definitions to preserve the integrity of participant storytelling. 6. Producing the Report: The final themes were woven into a coherent analytic narrative, supported by representative quotations. Comparative insights between FGCS and SGI subpopulations were integrated to address RQ2, particularly around cultural expectations and mental load. Reflexive commentary ensured transparency in how interpretations were reached.

The inability to conduct post-analytic member checking represents a limitation of this study. To enhance analytic rigor, two independent coders analyzed the data. Each coder completed the full coding cycle separately, and discrepancies were resolved through consensus meetings. This process established inter-rater agreement, strengthening credibility and reducing single-researcher bias. Cross-validation was also employed; both coders independently reviewed theme structures and confirmed their grounding in the dataset. Reflexive memos and analytic audit trails were maintained to support methodological transparency. Trustworthiness refers to the credibility, dependability, confirmability, and transferability of the research findings [27,28]. Credibility was established through prolonged engagement, including repeated reviewing of the transcript and participants’ quotations within context. Dependability was established through a detailed audit trail, including a description of analytic decisions, iterative coding notes, and reflexive journaling. Confirmability was established through reflective notes during data analysis, ensuring that interpretations were grounded in direct quotations from participants, thereby mitigating the researchers’ bias [29]. Transferability was established through providing a clear, detailed description of the research setting, a clear presentation of the study design, sample characteristics, and study procedure [21,27]. The authors are grounded in mental health and acculturation research and are both experts in qualitative research, which may have influenced the interpretation of culturally grounded narratives. However, they both maintained bracketing and ongoing reflexive discussions throughout the study, which helped to mitigate interpretive bias.

## 4. Results

The two resulting themes derived from data from Q1 are “*Finding self*” and “*Balancing Competing Demands*”. “*Finding self*” focused on familial tension, which was described by participants as the strain of seeking and asserting their autonomy and identity separately from their parents. Codes such as “doing what is best for me,” “what I want,” and “self-fulfillment” were encompassed in this theme. Interestingly, participants did not feel cultural restraints but seemed to embody culture as a part of their identity. Rather, there was an emphasis on being one’s own person apart from the familial unit. For FGCS participants, cultural restraints were not described as limiting; however, SGI students specifically reported culturally based academic pressure. Themes and associated codes are presented in Table 2.

“*{I am} trying to separate myself from my family as well as learn who I am and break against generational setbacks*”

*(FGCS, 18-yr-female)*

The participant’s statement about “generational setbacks” underscores prolonged barriers, such as the absence of parental college knowledge, limited access to academic role models, and family financial constraints, which shape participants’ sense of starting “behind” their peers, rather than the generic adolescent struggles.

“*[I’m struggling with] Fear of failure based on what I have been told is being successful*”

*(FGCS, 19-yr-female)*

“*Right now I feel like I’m balancing and forming my own identity.*”

*(FGCS, 18-yr-female)*

“*[I’m struggling with] making sure my major is right for me*”

*(FGCS, 18-yr-female)*

In the pursuit of autonomy, participants expressed frustration with the limited prior knowledge within their social and familial networks, which forced them to develop self-sufficiency and self-reliance. Examples stemmed from devoting significant time to understanding the system of higher education while navigating coursework.

“*Although I talk to friends about what I am going through, they don’t know any more than I do. That type of support system is like coping, it’s not a solution*”

*(FGCS, 19-yr-male)*

“*Family just expects you to figure it out because they can’t really help out and I still have to stay on top of my classes. Something like filling out FASFA was so hard and time consuming. I didn’t know what I was doing.*”

*(FGCS, 18-yr-female)*

The theme of “*Balancing Competing Demands*” underscores the multiple pressures students manage simultaneously: academic workloads, financial strain, work obligations, family expectations, and mental health needs. Although participants recognized the importance of mental and emotional well-being for achieving success, they often perceived it as an additional burden or task on their already long to-do list.

“*Currently my biggest struggle is managing stress, school, and on top of that my mental health altogether*”

*(FGCS, 18-yr-female)*

“*My current struggle is school and work life balance, then mental health*”

*(FGCS, 18-yr-female)*

“*I’m trying to balance health and exhaustion*”

*(FGCS, 18-yr-male)*

“*I can’t work because of my classes, but I need money. I need a job. It’s a lot*”

*(FGCS, 19-yr-male)*

“*I am working on time management, mental health, socializing*”

*(FGCS, 18-yr-female)*

“*I do not have a way to relieve my stress*”

*(FGCS, 19-yr-female)*

### Second-Generation Immigrant Students

Across the FGCS-SGI group, all of the participants referenced cultural expectations that shaped their academic stress. Five Hispanic-identifying participants described pressure linked to family sacrifice, often referencing parents’ migration journeys as the reason they “must not fail.” Two Asian-identifying participants emphasized career prestige norms (e.g., medicine, law, engineering). Afro-Caribbean participants (*n* = 3) discussed the expectation to “represent well” and uphold family honor. Although all SGI students described cultural expectations, the form and intensity varied by cultural background, showing that these pressures are neither uniform nor consistent. Fear of disappointing family members was mentioned by nine SGI participants, often related to worries that choosing a nontraditional major could be seen as wasting their family’s investment and sacrifice. From the dataset, the SGIs expressed the same sentiments in previously mentioned themes as their single-identity peers, but also conveyed the weight of having to meet and uphold specific cultural expectations regarding academic pursuits and career trajectories. This theme of Cultural Expectations was generated from Q2 codes. Moreover, feelings of perceived familial rejection due to failure combined with the pressure to succeed caused increased stress and mental load.

“*My stress truly began when I was a teenager transitioning to a young adult because I realized what I wanted out of my life and it did not align with my mom*”

*(FGCS-SGI, 18-yr-female)*

“*I think American culture gives more freedom, give more like allowance for things that are less practical like pursuits….not as practical like doctor and lawyer careers.*”

*(FGCS-SGI, 19-yr-female)*

“*There is pressure of being the best and succeeding at everything that you do*”

*(FGCS-SGI, 18-yr male)*

“*So now you like in between making your parents happy or making yourself happy*”

*(FGCS-SGI, 18-yr-female)*

“*Some of the restrictions that I continue to put on myself when I entered college [because] a little bit fear of my parent’s rejection*”

*(FGCS-SGI, 18-yr-female)*

## 5. Discussion

This discussion is organized according to Bronfenbrenner’s socio-ecological model [15], focusing on the individual, micro-, meso-, exo-, and macro-level factors that shape FGCS and SGI students’ mental health experiences. This facilitates understanding the gap of compounded mental health stressors within these intersecting identities. Story Theory guided both data collection and interpretation, illuminating small, everyday stories that reflect moment-to-moment meaning-making, emotional burdens, and identity negotiation. In accordance with the study aims to explore how FGCS and SGI intersecting identities are influenced by mental health challenges, the study methodology allowed this population to connect with themselves and peers through intentional dialogue guided by the tenets of the Story Theory. Viewing FGCS and SGI mental load through a multi-layered SEM lens helped to guide students toward creating ease.

### 5.1. Individual Level

Bronfenbrenner’s socio-ecological model shows the networks of people, communities, and overall policy influences that impact an individual by placing the individual at the center of the model, surrounded by concentric circles representing microsystem interactions between systems, the mesosystem (interactions between microsystems), the exosystem (indirect environmental influences), and the macrosystem (cultural and societal context) [15]. The individual characteristics are important, as this is where the model begins. There is significant overlap between FGCSs and the SGI population. Both groups face financial hardships, cultural mismatches, lack of social capital, and mental health challenges when pursuing higher education. Even if not from low-income families, FGCSs generally have a lower socioeconomic status [30], and SGIs, being children of immigrants, can recall or have experienced their families’ struggles to establish stability in a new country. The U.S. immigrant population is very diverse and can face several challenges during and after immigration, such as acculturative stress, poverty, limited English proficiency, housing insecurity, declining health, and discrimination regardless of immigration status [31,32]. This population experiences layered stress just by being born into an immigrant family. SGIs are disproportionately exposed to risk factors known to contribute to mental health disparities [8]. This cultural aspect and personal lived experiences add a deeply rooted stress component to the pursuit of higher education, as it is often viewed as a way to achieve upward mobility. Furthermore, these students recognize, acknowledge, and manage this stress while trying to stay true to themselves and remain culturally appropriate [9].

Yet these students possess several strengths that manifest in their ability to be resilient and overcome obstacles, as well as their resourcefulness, motivation, and persistence in seeking support from institutional agents [3]. It is possible that students who identify as FGCS-SGIs have learned from their parents the tenacity required to overcome difficulties and push towards a better future that can be visualized before it is actualized. This is evidenced by research indicating that immigrants, as well as their children, have higher levels of postsecondary educational attainment than natives [33]. Furthermore, post-secondary persistence is greater among immigrant and second-generation Black youth, which is especially evident in elite institutions [34], and this persistence is seen across racial ethnic children of immigrants [33].

What might be interpreted as “resilience” in prior studies may, in practice, represent participants’ necessity-driven persistence rather than a conscious sense of strength. This distinction is important because resilience narratives can unintentionally mask structural constraints, shifting responsibility onto students rather than the institutions meant to support them [35]. Social constraints refer to pressures that limit individuals’ ability to express distress or seek support, often driven by cultural norms of silence, perfectionism, or stoicism. These constraints can lead students to avoid effective coping strategies, mistakenly equating avoidance with resilience [36]. This interpretation aligns with participants’ perceptions of their mental health as an extraneous or secondary concern. Investing time in developing coping strategies may be regarded as non-time-sensitive and therefore less urgent than academic achievement, family expectations, and financial obligation. Over time, this prioritization may unintentionally cultivate patterns of avoidance and persistence toward visible accomplishments, which are emphasized at the expense of emotional well-being.

Although participants did not report clinical symptoms, they described emotional exhaustion, fear of failure, and chronic stress experiences consistent with subclinical distress patterns identified in the literature [37,38]. In our data, students commonly described emotional exhaustion, lack of coping strategies, feelings of being overwhelmed, and perceiving mental healthcare as burdensome, which indicates limited psychological bandwidth rather than resilience. Therefore, while existing research documents strengths in these populations, our findings suggest that “resilience” may coexist with unaddressed stress, avoidance, and cultural pressure, which require more nuanced interpretation. Within this context, identity development (“finding self”) involved both internal meaning-making and, particularly for SGI students, ongoing negotiation of culturally based academic expectations and family duties, rather than these elements being collapsed into a single construct.

Important differences emerged between the FGCS-only group and the SGI group. The theme “Finding Self” captured this overarching identity work across participants but took different forms in each subgroup. For FGCS-only students, “Finding Self” centered on navigating academic overload, lack of guidance, and constructing a sense of self as a college student without strong perceived cultural restraints. While FGCS participants focused on academic overload, lack of guidance, and navigating systems independently, SGI participants also stressed cultural expectations, parental pressures, and fears of disappointing family members. For SGI students, then, “Finding Self” unfolded under culturally based academic pressure and moral obligation, making familial tension a context for, rather than a replacement of, identity development. These cultural factors intensified their mental load and added a moral dimension to academic decisions, setting them apart from their peers who solely identified as FGCSs. Within the SGI group, cultural expectations varied according to cultural background. Furthermore, taking time to acknowledge mental health can be viewed as a lack of resiliency. This internalized perspective underscores cultural narratives and stigma of perseverance that equate help-seeking with weakness. Among SGIs, the link between immigrant status and mood/anxiety disorders was significant for racial/ethnic minority immigrants, and SGIs were more likely to be diagnosed with borderline personality disorder than native-born Americans [39]. Social constraints were also associated with higher levels of psychological distress among Hispanic/Latino and Asian college students [36].

### 5.2. Microsystem (Interpersonal)

Microsystems are smaller communities with which the FGCSs and SGIs interact frequently, shaping their worldview and higher educational experiences. Micro communities can include family, social and academic friend groups, work, classrooms, clubs, and organizations. People within these micro communities can create various parallel experiences, with the FGCSs and SGIs serving as the intersecting agent. Due to the bi-directional nature of interactions within micro-communities, the intersecting agent (FGCSs and SGIs) can both benefit from and provide benefits to these micro-communities. As the intersecting agent moves amongst these communities, there is the benefit of learning and growing from a myriad of experiences and bringing that knowledge back to assist others who may benefit. Although this is a form of community and familial upward mobility through education, it can also impact the mental health of the FGCS-SGIs. The FGCS-SGIs must engage with various aspects of their personal, cultural, social, and academic identities as they navigate through these micro-communities. When aspects of the dominant culture conflict with one’s cultural identity, it may lead to greater self-focused thoughts and experiences of cognitive dyscontrol [40].

Within familial and social micro-communities, FGCS-SGIs can forgo formalities and find social support, although it may not help alleviate the challenges encountered in other micro-community settings. Many of our study participants described their social and familial relationships as a source of coping rather than a way to learn and/or find solutions to their various challenges. Participants described familial and peer support as mainly emotional rather than practical, without acknowledging that emotional coping remains a valid and meaningful form of support. Specifically, citing that their friends provided no additional knowledge revealed frustration with emotional support due to a lack of instrumental knowledge. FGCSs wanted solutions and pragmatic responses to their questions and associated stressors, not abstract conceptual overviews that required additional independent investigation on the part of the student. With instrumental needs being at the forefront of their minds, FGCSs appreciated but undervalued this emotional support reinforcing FGCSs viewing their mental health as a nuisance rather than a component of their well-being.

FGCSs need instrumental guidance (e.g., navigating FAFSA, understanding academic policies), which families and close friends might be unable to provide. Instead of attributing this solely to shortcomings within FGCS-SGI immigrant families, it is important to recognize their strengths, collectivism, sacrifice, and interdependence while also acknowledging structural barriers that limit their ability to offer academic guidance. Often, these students expressed frustration when attempting seemingly routine and mundane tasks (e.g., completing financial aid forms), which further highlighted their lack of capital within the higher education arena. This reflects a recurring tension between emotional comfort and instrumental support in the FGCS-SGI immigrant family system.

### 5.3. Mesosystem—Collection of Microsystems (Organizational)

The university or higher education mesosystem encompasses various micro communities, including classrooms, assistive institutional offices, clubs and organizations, work–study programs, and friendship circles. Participants did not directly mention the university structure beyond workload and navigating university offices. Yet when considering the university mesosystem and the impact of professors on students, the authors feel it is important to consider the application of theory to the faculty role within the FGCS and SGI mental health context. By cultivating and nurturing an environment that allows students to express their personal circumstances, educators acknowledge the wholeness of the individual beyond academic performance. Incorporating activities that recognize and appreciate a student’s personhood, such as providing opportunities for self-expression, inviting students to share their preferred learning style, or integrating culturally sensitive practices, can affirm their dignity and enhance a learning space grounded in empathy, respect, and inclusion. Lastly, students interpret basic levels of faculty care and concern as an indicator of not only belonging but also the importance of their academic journey, not only to them but also to the educator, which is essential to students feeling accepted and affirming their whole self belongs in the academic space [3]. Within this intersection of identity, caring faculty play an integral role in academic progress and students’ holistic well-being, reflecting as co-journeyers who share in the burden and triumph of student success, which can help lessen the mental burden. This application of the literature aligns with Story Theory’s idea of intentional dialogue, where supportive relational encounters help individuals articulate their concerns, recognize alternative meanings, and experience emotional relief [13].

Conducting check-ins with students at the beginning or end of class, such as asking them to identify one thing that is clouding their mind or providing an opportunity for each student to schedule a time with the educator, further embodies authentic presence, connection, and trust, which helps dismantle social constraints. When students feel that faculty and staff care for their well-being and provide holistic support, they can ask questions, are connected to resources, and can problem-solve with a faculty or staff member [3]. When faculty, staff, and peers demonstrate care, they cultivate inclusive and humanistic cultural norms for FGCSs with multiple stigmatized identities [3]. Although the university mesosystem can consist of mental health counselors and psychologists, professors are the first-line interactors with the student body.

### 5.4. Exosystem and Macrosystem—Negative Social Rhetoric and Immigration-Related Policies

The exosystem level includes social structures and programming, both formal and informal, that indirectly affect both FGCSs and SGIs, even if the individual has no direct interaction with these structures. The macrosystem refers to the broader cultural and societal context that impacts how all other systems function [15]. For many SGI students, sociocultural forces such as xenophobia, racism, discrimination, and stigma surrounding mental health create an additional layer of emotional strain. Although participants did not explicitly reference national policies, they described being affected by community narratives, family conversations, and media portrayals of immigrants, which shaped their perceptions of safety, belonging, and access to support. These broader societal pressures may heighten vulnerability to anxiety and depressive symptoms among racial/ethnic minority SGI students, consistent with prior research [40]. SGIs can gain exposure to all these factors through their neighborhoods, familial conversations, and news and media output, which can affect their view of supportive structures and personnel. Although it can be stated that these policies are not directed at United States-born citizens, the literature reveals that U.S. citizens in mixed-status families also reported hesitancy in accessing healthcare, including mental healthcare, out of concern for their loved ones [41]. Policy-level changes at the community and educational levels are necessary to help address many aspects of mental health challenges.

Story Theory guided both data collection and interpretation by emphasizing small, everyday stories as meaningful insights into participants’ lived mental load. This theoretical perspective encouraged attention to fragmented, evolving, and “messy” stories rather than seeking fully resolved accounts. As a result, the themes reflect ongoing identity negotiation, emotional burdens, and moment-by-moment meaning-making characteristics of small story narratives. Story Theory also influenced the analytic approach by valuing co-constructed meaning during the focus groups, aligning with the dialogic nature of participants’ exchanges. Important differences emerged between the FGCS-only group and the SGI group. While FGCS participants focused on academic overload, lack of guidance, and navigating systems independently, SGI participants also stressed cultural expectations, parental pressures, and fears of disappointing family members. These cultural factors intensified their mental load and added a moral dimension to academic decisions, setting them apart from their peers who were solely FGCSs. Within the SGI group, cultural expectations varied according to cultural background.

## 6. Limitations

Although focus group discussion has the potential to generate rich, robust, and interactive small stories as seen in this study, it may have constrained the expression of participants to some questions. Some participants may have withheld their perspective due to the presence of peers or concern about judgment. Although focus groups help to generate discussion, it can also limit the depth of the data from an individual perspective. Notwithstanding, the focus group facilitator endeavored to ensure a conducive environment to enable participants to tell their stories. It is possible that only those genuinely interested in the topic chose to participate in this study, which limits the generalizability of the results as students willing to discuss mental health may differ from those who avoid these topics, potentially underrepresenting the most distressed individuals. Also, due to data being collected in the Office of First-Generation Student Success at one institution, the results may not fully capture how mental health beliefs and attitudes develop throughout their college experience. Furthermore, future research should investigate the mental load of this population at institutions without supportive offices. Lastly, nine participants were male; thus, examining gender differences in FGCS and SGI mental health experiences can provide great insight in future studies.

### Future Implications

Findings from this study suggest that FGCS and SGI students encounter unique challenges in accessing and engaging with mental health services, shaped by cultural beliefs, family expectations, and personal experiences. Future research should investigate long-term changes in mental health beliefs and coping strategies throughout college, also accounting for the male perspective. To address these challenges, institutions should prioritize the development of culturally safe mental health services that transcend cultural competence to assist students with navigating intersecting marginalized identities. Culturally safe care encompasses practical measures, such as employing bilingual and bicultural counselors, utilizing therapy models that incorporate family participation when appropriate, and training clinicians to recognize culturally shaped expressions of distress (microsystem). Additionally, it involves counseling approaches that avoid invalidating students’ cultural identities or obligations (individual level). Institutions can take immediate steps by implementing culturally responsive mental health programs such as peer-led support groups, culturally competent counseling services, and awareness campaigns tailored to the various experiences of FGCSs and SGIs (mesosystem). These efforts should aim to create safe, inclusive environments where students feel comfortable seeking help without fear of stigma. Additionally, policy-focused research (macrosystem) is necessary to examine how institutional policies and campus resources influence mental health-seeking behaviors among multi-generational immigrant populations. This finding necessitates a shift from siloed mental health services toward integrated academic–mental health models, such as embedding mental health counselors within academic advising units or offering course-based wellness interventions (mesosystem). While community-based support groups may still be beneficial, the primary implication of our data is the need for institutionally integrated support, not standalone services.

## 7. Conclusions

This study highlighted how intersecting identities of FGCS and SGI students shape their mental health challenges, illuminating how these students make meaning of their challenges through intentional dialogue aligned with Story Theory and operationalized using socio-ecological models. Clear distinction emerged between the FGCS-only group and the SGI group. While FGCS participants focused on academic overload, lack of guidance, and navigating systems independently, SGI participants also stressed cultural expectations, parental pressures, and fears of disappointing family members. These cultural factors intensified their mental load and added a moral dimension to academic decisions, setting them apart from their peers who solely identified as FGCSs. Within the SGI group, cultural expectations varied according to cultural background. For example, Asian-identifying participants emphasized achievement and career prestige, while Hispanic students highlighted family sacrifice and obligation. Afro-Caribbean participants described feeling pressure to “represent well” as the children of immigrants. Although all SGI students experienced cultural expectations, the form and intensity differed. To stay aligned with our findings, the discussion primarily focused on themes emerging directly from the data, using existing research only to provide context, not to overshadow participants’ lived experiences. Beyond adding to academic knowledge, this study highlights the urgent need for institutions to adopt culturally tailored mental health support and proactive engagement strategies. By taking deliberate and informed actions, universities can lower barriers, promote fair access to care, and support the psychological well-being of their diverse and growing student populations.

## 8. Positionality

The first author identifies as an FGCS-SGI. This identity was communicated to all participants at the beginning of each focus group discussion. This author deliberately asked participants to elaborate on their thoughts so as to not infer with participants’ stories. This author kept a reflexive journal to assist with reflection and separation of researcher bias. The second author, who identifies as an African immigrant scholar with a nursing background, acknowledges that his professional and cultural standpoint may influence how he interprets participants’ narratives, particularly in relation to issues of adaptation, resilience, and identity. To ensure analytic integrity, he engaged in ongoing reflexive dialogue with the first author, bracketed personal assumptions during data analysis, and consistently grounded interpretations in participants’ voices rather than his own experiences. This collaborative reflexivity strengthened this study’s credibility and ensured that the analysis remained focused on the meanings constructed by the participants themselves.

## Figures and Tables

**Table 1:** Demography of sample population.

Demographics	*n*	Cumulative %
**Age**		
18 years old	18	82%
19 years old	4	18%
**Gender**		
Female	13	59%
Male	9	41%
* Cultural Identification		
American	11	50%
Hispanic	6	27%
Asian	1	4.5%
Bangladesh	1	4.5%
Afro-Caribbean	3	14%
**Majors**		
STEM and Health Sciences	9	41%
Marketing	3	14%
Political Science	1	4.5%
Communications	2	9%
Business	1	4.5%
Psychology	2	9%
Hospitality	1	4.5%
Education	1	4.5%
Finance	1	4.5%
Undecided	1	4.5%

* Cultural Identification reflects how participants self-identified their cultural affiliations.

**Table 2:** Codes and overarching themes.

Overarching Themes	Codes
Finding Self	Doing what is best for me What I want Self-fulfillment Understanding who I am Depending/trusting myself Is this what I should be doing Learning myself apart from parents Identity Formation Learning on my own
Balance Competing Demands	Balancing multiple roles and responsibilities Not enough/too much personal time Difficulty focusing or relaxing/always tired Don’t have time to care about stress It’s a lot Time management, exhaustion, fatigue
Cultural Expectations	Cultural responsibility Acknowledging/Honoring parents journeys and sacrifice Don’t embarrass family/Don’t let family down Fear of failing family

## Data Availability

The data presented in this article is not readily available because it is part of an ongoing study and also due to privacy and legal considerations for participants. Requests to access the data should be directed to the corresponding author.

## Supplementary Materials

The following supporting information can be downloaded at: https://www.mdpi.com/article/10.3390/healthcare14010021/s1, Semi-structured Interview Guide.
