# Supplementary material for: Mental Health Challenges at the Intersection of First-Year, First-Generation College Students and Second-Generation Immigrant Identities: A Qualitative Study
Source: Healthcare (Basel). 2025 Dec 21;14(1):21. doi: 10.3390/healthcare14010021 (PMC12786242; doi:10.3390/healthcare14010021)
Supplement: Supplementary file 1 [file healthcare-14-00021-s001.zip › healthcare-3986101-supplementary.pdf]

Semi-structured Interview Guide

What does being a first generation college student mean to you?

What is your experience of being a first-generation college student?

What is your cultural background?

What does being a second-generation immigrant mean to you?

How would you describe your culture?

As you consider your future, are there any other factors that you think about besides your own desires?

If so what are those factors?

How does that make you feel?

Have you experienced stress, anxiety, or depression related to your background or identity?

Can you describe a time when you felt your mental health was affected by your identity (for instance, being a first-generation student or a second-generation immigrant)?

What currently weighs you down mentally and/or emotionally?

What is currently causing you the most mental load or stress?

How do you "manage" your mental health and well-being?

What do you do for rest and relaxation?

Do you have support?

What does support look like for you?

What coping strategies or support systems have helped you deal with these challenges?

What kinds of support are most helpful?

What advice would you give to other students navigating similar intersections of identity?

Is there anything we didn't discuss that feels important to share?

How was the experience of talking about these topics for you today?

Thank you for your participation in this study
